# A Woman’s Right

**Published:** 1873-01

**Authors:** Ben. H. Pratt


### A WOMAN'S RIGHT.

BEN. H. PRATT, A. M.

He who lives without an occupation or business must give the world a good reason for it. She who engages in any business or occupation by means of which money is earned, must likewise render unto the world a good excuse therefor.

This is a law of Society. Society rigorously enforces this law. Society is a good ruler, but a bad legislator.

Without departing one iota from that proper veneration for the female sex, which of itself is the grand dividing line between civilization and barbarism, I cannot but raise an opposing voice to the extreme to which this law is carried.

So long as the world turns there must be grades of Society, but Caste is contemptible.—Conditions of men must vary. Refinement and culture, especially in our own country, may exist as well in the homes of the humble as in the mansions of the great. Society, then, if wise, should not enact a law which can be obeyed by one class without effort, while the other cannot obey without suffering for it.

What I want to get at is this: why may not the lady of refinement and culture and intelligence and accomplishments, enter upon any respectable and appropriate employment, sufficiently remunerative to make her independent of others? That is, provided she has no just claims upon those who are perfectly able and willing to provide her with all her tastes demand.

You and I know that she can't do this now, and hold a position in Society. All will praise the lady who makes a heroic effort to sustain herself—call her a heroine—admire her pluck—encourage her in her undertaking—but she has stepped without the pale of intimacy with her former associates. They meet her with the same smile—the same kiss—but not with the same feeling. She and they now bow down to different shrines—hers is duty—theirs, inclination. There is now no longer any unanimity of thought and feeling and tastes. She is a friend, but not so particularly so as aforetime. There is not so much haste to introduce her to a visitor—not that eagerness to have a stranger friend know her—she is day by day getting further removed from her former status—by and by she is dropped.

This "dropping" is what hurts. If my reader has ever been bold enough to defy Society in the way I have mentioned, she will know just what it means to be dropped. True, she falls into another sphere, but even companionship in misery is not so delectable as the adage would have it.

Why, again, must it be a disgrace, in a modified form, for a lady to work *for pay*? I know ladies right on the very peakedest peak of the peak of Society, who will put their servants to shame in kitchen work—but not for pay. Others will do miracles of sewing machine work for themselves or friends—but not for pay. Others still will stand behind the counter at a "ladies' fair" and use all manner of wheedling tricks to catch a customer—but not for pay. Still others will even assume the role of the beggar, and don't disdain to extend an eager palm for pence to help along a favorite missionary hobby.

Where's the difference?—Pay. One must; the other need not. Tweedledum and tweedledee.

How singular it is that prejudice is so much stronger than good sense. Have you not often felt your inability to give a good sensible idea a place in your mind?

On one side of the street a lady is leisurely wending her way to a friend's. She is arrayed in fine apparel she never earned—jewelry she didn't pay for—her whole style, the result of somebody's hired taste.

On the opposite side another lady is briskly going to business. She wears modestly a modest amount of dress which she has earned herself and don't make any fuss about.

Very strangely, we stop to admire the one and ignore the other. We wouldn't if we were sensible, but then we do. When, however, a streak of good common sense comes over us, we calculate the price per yard of the velvet cloak ahead—the probable value of that fringe per foot—the number of pennyweights of gold in the ear drops—the sum the husband probably paid for the whole thing—and whether he didn't likely overdraw his bank account to pay his last milliner's bill. The walking exhibitor don't come in for even a thought, and might be a steam automaton for all we care for it.

This prejudice against women's working for pay is something mysterious. We all have it. It must be the result of an abnormal condition of Society, because it isn't right. Everybody admits that. How it came about is as explainable as the epizootic.

When we chaps want to escort a lady to the opera we don't go down to Lace, Trimmings & Co., and ask one of their lady clerks to honor us with her company. If we have an evening on our hands, we don't care to visit Lock Stitch & Bro.,—though we may find a higher class of conversationalists and a rare intelligence among the busy operators of that enterprising firm. But both of these would certainly be a sensible thing to do. Miss Astrakhan wouldn't think our taste very good, to be sure, nor would Madam Sableset honor us at her next little buzz; still one might exist, possibly, and not be wholly miserable thereafter.

Now see what harm our prejudices are doing to many. Here is a lady entirely dependent upon somebody for her food and clothing. She has no particular claim upon them, but she must live and move and have her being—which means, sit at a first class table, wear just as good clothes as any in "her set," and have pin money enough to do as they do. She don't like to be dependent—she groans in spirit every day—she's miserably blue over it sometimes. But, dear me, what can she do? She can't face employment, or they would read her out of "her set." Her moral courage is as great as the general run, perhaps, but it's a little too big a sacrifice to make, and so she keeps depending and feeding her unhappiness with what it grows on. She don't admire work any how—never did, for that matter—and as it isn't fashionable for women of position to labor, she concludes to continue a useless incumberance until something turns up.

Can work for women be made popular—or fashionable, rather? That's a conundrum which most of us give up.

Wouldn't it help matters a little if every mother required her daughter to learn to do everything about a house, for instance? Not to learn it theoretically, as some do, and then boast of it, but become proficient by constant practice. There is so much variety about household duties, that I really think that no young lady brought up to a perfect familiarity with all its details, need learn anything else in the way of work, to secure herself against misfortune, should it come, or against dependence, were she brought to that.

If these be distasteful, as they are to many, let the piano be mastered instead of being used as an occasional entertainer; or a language learned so effectually that the learner may become the teacher if need be; or if artistically inclined, let painting be learned instead of played with. Teaching the younger members of a family is especially the province of the older, and if carried to a proper extent, is the best school of the future teacher.

When every child is firmly impressed with the idea that he or she is expected to perform some special work in the household economy—is assigned to the superintendance of some of its departments, for the proper fulfilling of the duties of which it is wholly responsible—an important step in the formation of that child's character is taken. As years advance, more and more responsibilities should be placed upon children: at maturity the whole burden of housekeeping should rest upon the shoulders of the daughter, or if more than one, upon each in turn for a period of years.

It is this foolish method of relieving the daughter of all care—of administering to her wants and expecting nothing in return but gratitude—of affording her access to every source of enjoyment, and removing from her as much as possible, the vexations of self help and self reliance, that is poisoning the Society of the present day.

What wonder it is that young ladies consider work a burden, nay, even a disgrace, when they are so taught from infancy, and see the whole onus of household labor put into the hands of hirelings. Not only pernicious in its moral effects, is such a system, but preeminently so in a physical sense. Labor is the natural stimulus of healthful life. Without it our lives become an aimless sham. We must have work to do [illegible] we must have air to breathe. All are given capacities for work—divine injunction sanctions it—example proves its necessity. The great workers are the great thinkers, attesting the fact that work is the proper aliment for the mind—the safeguard of the thoughts.

The woman question of to-day is unpopular only by reason of its inconsistency. Ambition overtops reason. The reformers begin at the wrong end. We are to believe that woman may be President, Senator, Judge, before she has served her apprenticeship in the lower ranks of life. When it is popular for women to enter upon professions, and other occupations as men do, by beginning at the lowest rung of the ladder and working up—when they may range themselves side by side with men in any business for which they are adapted, and meet defeat and success as men do—when it becomes no disgrace for a woman to work for her living—then the woman question will be in a fair way of amounting to something practical, and therefore worth consideration by the combined intelligence of the nation.

Woman is not a divinity—to be worshipped. She is not an angel—to be adored. She is not too good to work—nor too ethereal to contend – nor too weak to be trusted—nor too delicate to be independent. Devotion to the sex does not consist in spoiling them—making of them useless dolls and lay figures for the parlor and beautiful statues vivants for our friends to step in and admire.

This is too true to be ridiculous but not too ridiculous to be true. We all know men who boast of their daughters as they do of their mansion, their furniture, their statues, paintings and other articles of vertu. The uselessness, the unfitness for practical life, the emptiness, the silly clap-trap superficiality of the daughters of wealth and luxury, are proverbial. Men of brains and no means, shun them. Men of means and no brains endure them. Society winks at the game of match and catch and posterity suffers in consequence.

And then it isn't the fault of the women perhaps, after all, any more than that of the men. There is a tendency to debar females from work even appropriate for them. There is a dislike on the part of almost every one to have his sister or cousin or intimate friend admit, first the necessity for labor, second the unwillingness of friends to support them without labor.

So soon as the labor of woman does not deprive her of the Society for which she is fitted intellectually—so soon as work is loved for work's sake, and not regarded as appropriate for menials only—so soon as men can see the worth of woman's work—so soon as it will not reflect upon us to have our sisters engaged in some respectable calling—then, and not till then will woman's independence begin, her self respect increase, and her self reliance become something to be proud of; and an added conciousness that she is a useful as well as an ornamental adjunct of the social economy will obtain.

